# Object use in communication of semi-wild chimpanzees

**DOI:** 10.1007/s10071-023-01792-z

**Published:** 2023-06-14

**Authors:** Violet Gibson, Sarah T. Boysen, Catherine Hobaiter, Marina Davila-Ross

**Affiliations:** 1 Centre for Comparative and Evolutionary Psychology, University of Portsmouth, Portsmouth, PO1 2DY UK; 2 Comparative Cognition Project, Sunbury, OH USA; 3 School of Psychology & Neuroscience, University of St Andrews, St Andrews, KY16 9JP UK

**Keywords:** Object use, Chimpanzees, Communication, Social interactions, Tool use

## Abstract

Object interactions play an important role in human communication but the extent to which nonhuman primates incorporate objects in their social interactions remains unknown. To better understand the evolution of object use, this study explored how objects are used in social interactions in semi-wild chimpanzees (*Pan troglodytes*). We used an observational approach focusing on naturally occurring object actions where we examined their use and tested whether the production of object actions was influenced by the recipients’ visual attention as well as by colony membership. The results show that chimpanzees adjusted both the type of object used, and the modality of object actions to match the visual attention of the recipient, as well as colony differences in the use of targeted object actions. These results provide empirical evidence highlighting that chimpanzees use objects in diverse ways to communicate with conspecifics and that their use may be shaped by social factors, contributing to our understanding of the evolution of human nonverbal communication, language, and tool use.

## Introduction

Humans often rely on objects to aid their communication, where references can be made to actions, events, and individuals (Park 1997). Such object interactions are important tools in human communication. They may be used to facilitate information exchange in children in the early stages of language development (Iverson 2010) and people with impaired or atypical communication (Jones et al. 2002; Manzi et al. 2020; Thiemann and Goldstein 2001), as well as to enhance explanations about complex topics (Hazel 2014). This area is, however, yet to be fully understood, especially from an evolutionary perspective, with research in humans primarily exploring language and one-to-one interactions without object use (Williams and Kendell-Scott 2006). The role of objects in social contexts is often overlooked, particularly their use in nonhuman primate species’ communication. To shed new light on this topic, the aim of this paper is to explore how chimpanzees use object actions when interacting with others and examine the effect of social factors on object signalling. Understanding what features of human communication are shared across modern ape species is key to our ability to trace its evolutionary trajectory. Extending our focus to include object-based social interactions allows us to consider alternative means of communicating and how these may have supported the development of nonvocal communication.

Descriptions of object use in nonhuman ape signalling include object play (Myowa-Yamakoshi and Yamakoshi 2011) and the use of objects to modify the acoustic form of vocal signals (Peters 2001). However, the majority of work has focused on accounts of object-use in gestural communication; predominantly in chimpanzee communication (Fröhlich et al. 2017; Hobaiter and Byrne 2011a; Liebal et al. 2004a; Nishida et al. 1999; Tomasello et al. 1994). Gestures are important facilitators of nonvocal communication, often closely woven in with other communicative signals such as vocalisations, facial expressions, and gaze (Hobaiter et al. 2017; Leavens et al. 2010; Parr et al. 2016; Wilke et al. 2017). They support complex social interactions and regulate relationships among conspecifics (Arbib et al. 2008; Maestripieri 1999). In some cases, empty-handed gestures are modified by adding a physical object, for instance as in hitting or hitting with object, poking or poking with object (Fröhlich et al. 2017; Hobaiter and Byrne 2011a; Liebal et al. 2004a). Other object-gestures exist as independent forms without a non-object counterpart, for example: leaf clipping, slapping object, moving object, shaking object, waving object (Graham et al. 2018; Hobaiter and Byrne 2011a; Nishida et al. 1999) or spitting or splashing water (Hobaiter and Byrne 2011a; Tomasello et al. 1994). The inclusion of objects in gestural communication adds another element to a system that is already considered highly diverse and flexible (Call and Tomasello 2007). However, the extent to which object use occurs in social interactions outside of gestural communication remains largely understudied. In addition, it is currently unclear whether social factors known to impact nonhuman primate communication would also shape social object use more broadly. Building on these initial reports of communicative object use, the first aim of this study was to explore the use of object actions in chimpanzees and their flexibility across contexts. Here we consider flexibility to represent ability to produce adjustments to various communicative circumstances, such as different behavioural contexts. We then examined the effect of social factors on the production of object signals including the recipient’s visual attention and the subjects’ colony membership. Previous studies have identified the flexible use of signals across contexts, and the adjustment of signal modality to match a recipient’s ability to perceive them as key indicators of sophisticated cognitive abilities (Freeberg et al. 2012), and foundational capacities for highly flexible forms of communication such as language.

Numerous studies have investigated these elements including the degree to which a single signal functions across different contexts (Hebets et al. 2016). Great ape gestural repertories are highly flexible (Call and Tomasello 2007; Genty et al. 2009; Liebal et al. 2006; Tomasello et al. 1994), which is thought to enhance communicative efficiency (Snowdon 2008), for example: by allowing the interaction between context and signal to influence the response of the recipient (Roberts et al. 2012). While some gestures appear to be more flexible than others (Hobaiter and Byrne 2014), there is some evidence that object-based gestures may also be used flexibly across contexts (Fröhlich et al. 2016; Hobaiter et al. 2017). For example, chimpanzee object-gestures such as throw object, stomp on object, and wave object were observed in several contexts relating to access to infants, aggression, play, and sex/courtship (Liebal et al. 2004a; Nishida et al. 1999). Similar observations were also made in orangutans (throwing, showing, and shaking: Liebal et al. (2006)) and gorillas (throwing, drumming, and punching: Genty et al. (2009)), but the extent of such reports remains largely restricted to gestural signals.

Because of the interest generated by the discussion that ape gestures, like human language, are used with intention, that is they are targeted towards a specific recipient in order to achieve a specific goal, studies of gesture have typically employed restrictive definitions that require these signals to meet criteria for intentional communication (for example response waiting, persistence, or elaboration Byrne et al. 2017; Genty et al. 2009; Leavens and Hopkins 1998, 1999). In doing so, these studies exclude body signals for which there is no clear evidence that the signaller intends them to be targeted. However, primate audiences are capable of decoding nuanced information within a rich range of signals, for example: extracting information on threat type and urgency from broadcast alarm calls (Crockford et al. 2012; Fischer 2020; Murphy et al. 2013; Wheeler 2010). As a result, limiting the study of object use in social interactions to object-based gestures, may limit our ability to fully describe how objects are incorporated in ape signalling. For this reason, we set out to examine communicative object use beyond solely object-gestures, incorporating object actions that may not necessarily adhere to the restrictive criteria  for intentional communication. Drawing from the current literature, we hypothesise that chimpanzees have the ability to flexibly use a range of targeted object actions across behavioural contexts, potentially enhancing the efficiency of their communication (H1: flexibility across contexts).

The choice of chimpanzee gesture is further modified by the visual attention of the recipient, particularly in its sensory modality: all gestures have a visual component, but others include tactile and auditory information (Genty 2019; Leavens et al. 2010). Apes’ sensitivity to their recipient’s attention is most easily recognised in the production of visual gestures, as visual attention – through gaze direction–is more easily detected than tactile or auditory ‘attention’. Silent-visual gestures are most likely to occur when the recipient is already visually attentive (Defolie et al. 2015; McCarthy et al. 2013; Pika et al. 2003), but where this is not the case signallers will typically move into a more suitable position, or select a gesture that includes an additional mode of information–such as touch or sound (Canteloup et al. 2015; Genty et al. 2009; Hobaiter and Byrne 2011b; Liebal et al. 2004b, 2006). Such sensitivity to others’ visual attention can already be observed in young chimpanzees who use silent-visual gestures more when interacting with a visually attending partner and produce tactile gestures more when the recipient is visually inattentive (Dafreville et al. 2021; Tomasello et al. 1994). Interestingly, research on the hypothesised attention-getting function of tactile and auditory gestures concluded that they are unlikely to be used as attention getters to facilitate further visual communication (Botting and Bastian 2019; Poss et al. 2006; Tempelmann et al. 2011; Tempelmann and Liebal 2012), and instead tactile and auditory gestures are employed to convey information independently, in a similar manner to visual-silent gestures (Hobaiter and Byrne 2011a; Liebal et al. 2004b).

Objects can be incorporated into signal use in a number of ways–providing visual-only (e.g. object wave), auditory (e.g. leaf clip, object shake), or tactile (e.g. hit with object) information. However, there has been limited exploration of the relationship between object interactions and visual attention outside of gesture. Most of the available evidence is based on food-requesting paradigms in which a chimpanzee needs to request food from a human experimenter. The impact of changes in the visual attention of the experimenter (for example facing towards or away, eyes open or closed) on the signal-use by the chimpanzee is then measured (Hostetter 2011; Hostetter et al. 2001; Kaminski et al. 2004; Leavens et al. 2004a, 2010; Tempelmann et al. 2011). Here, while an object is the focus of the interaction, it is not typically incorporated into communication by the signaller. We explore the effect of recipient’s visual attention on the use of objects by the signaller in social interactions. In addition to modality, the choice of object, specifically its rigidity, was also examined. Chimpanzees possess the ability to select tools on the basis of their physical properties, for example: rigid sticks are used to dig up termites, stiff twigs to open the termite tunnels, and finally flexible brush-tip fishing probes to extract the termites (Sanz et al. 2004). Building on the available literature on gesture use, we hypothesised that chimpanzees would adjust the modality of their object interactions, as well as object type, to the recipient’s visual attention (H2: flexibility to attention).

The already elaborate communication of nonhuman primates (Higham and Hebets 2013), can be further impacted by their social and physical environment (Graham et al. 2022), and their development and experiences (Bard and Leavens 2014; Leavens and Bard 2011; Snowdon 2009). This flexibility becomes particularly evident when exploring the role of social learning in communication and population specific behaviours (Janik and Slater 2000; Marshall et al. 1999; Mitani et al. 1992). Gestures such as pointing and clapping are often used in captivity to request food, behaviour rarely seen in wild apes (Cartmill and Byrne 2007; Leavens and Hopkins 1999), although hand clapping in wild lowland gorillas has been observed in vigilant and play contexts (Salmi and Muñoz 2020). However, despite this flexibility, the majority of apparent group differences in natural signal repertoires can often be explained as a result of sampling effects (Hobaiter and Byrne 2011a). The repertoires of vocalizations, facial expressions, and available gesture types – including object gestures–seem to largely overlap across groups and populations within a species (Bergman et al. 2019; Davila-Ross et al. 2009; Genty et al. 2009; Hobaiter and Byrne 2011a; Waller and Micheletta 2013), and, in their gestures, to a large extent across ape species (Byrne et al. 2017; Graham et al. 2018). Interestingly, this overlap in the available gesture repertoire stands in current contrast to the broad evidence available for socially-mediated group differences in material tool-use (Luncz et al. 2012) and foraging (Byrne et al. 2011; Hohmann and Fruth 2003; Jaeggi et al. 2010), and for social learning of non-functional object-behaviour (van Leeuwen et al. 2014). While the evidence remains limited in communication there has also been only limited investigation of the question (but c.f. Badihi et al. 2023), thus, it seems reasonable to hypothesize that chimpanzees may also learn socially about object interactions and as a result we predict that these interactions will differ between colonies (H3: group specific variation). Our findings will improve our understanding of the extent to which social groups of nonhuman primates may differ in their nonvocal signals, which have traditionally received less attention (Davila-Ross et al. 2011; Lameira 2013).

To test our three hypotheses, we examined object actions in 75 semi-wild chimpanzees living in three groups at Chimfunshi Wildlife Orphanage (CWO), Zambia. An Object Action was defined as the use of an object during any social interaction among chimpanzees. As a result, Object Actions were considered as a broad category of signals, which may have included but was not restricted to cases of gesture (Genty 2019; Graham et al. 2017; Hobaiter and Byrne 2011a; Liebal et al. 2006; Nishida et al. 1999). Similar to object-based gestures such as hitting (Hobaiter and Byrne 2011a) and throwing at (Liebal et al. 2004a), Targeted Object Actions were those in which the object appeared to be explicitly aimed towards or made contact with the conspecific. Nontargeted Object Actions such as object waving, holding/carrying, and splashing were also considered. While these were not targetting a specific conspecific, in all cases the apparent recipient was in close social proximity to the signaller, and was identified as engaging in ongoing social interaction. As multiple, isolated, chimpanzee groups live in the same forested environment in Chimfunshi Wildlife Orphanage, we were able to mitigate the impact of variation in genetic and ecological factors on variation in behaviour (Laland and Janik 2006) and could thus employ an ethnographic approach (Hamilton and Taylor 2017). Inter-group differences in communication have already been reported for these colonies (Davila-Ross et al. 2011). Expanding our research focus beyond the more commonly studied communicative signals can provide important insights about chimpanzees’ communication. If indeed, object-based signals are more extensively employed than previously recognised, nonhuman communication cannot be fully explained without addressing the role of objects.

## Material and methods

### Subjects and study site

Subjects were 75 chimpanzees living in three different semi wild colonies (Colony 1: N = 24 chimpanzees, Colony 2: N = 39 chimpanzees, Colony 4: N = 12 chimpanzees) housed at the Chimfunshi Wildlife Orphanage (CWO), Zambia. The individuals included in this study were 23 mature females, 10 mature males, 7 adolescent females, 6 adolescent males, 8 juvenile females, 10 juvenile males, 5 infant females and 6 infant males (see Table 1). We discriminated three age groups: mature individuals (10 + years old), juveniles (5–9 years old), and infants (0–4 years old) (Davila-Ross et al. 2011; Hobaiter and Byrne 2011a). All chimpanzees were members of multi-male, multi-female communities with the opportunity to express fission–fusion social dynamics. For the majority of the day (22 h) chimpanzees stayed outside in large outdoor enclosures. The remaining 2 h were reserved for indoor feeding time in smaller sub-groups to manage aggression.

**Table 1:** Overall sample composition with colony, sex group, age group, rearing history, and place of birth breakdown (N = 75)

	Sex		Rearing history			Place of birth			Subspecies		
	Male	Female	Mother / group	Hand raised	Unknown	Wild	Captivity	Unknown	Pan troglodytes schweinfurthii	Pan schweinfurthii	Unknown
COLONY 1											
Adult	5	8	6	6	1	6	6	1	6	2	5
Adolescent	2	1	3	–	–	–	3	–	1	–	2
Juvenile	3	3	6	–	–	1	5	–	1	1	4
Infant	–	2	2	–	–	–	2	–	–	–	2
COLONY 2											
Adult	2	13	4	8	3	9	3	3	7	3	5
Adolescent	2	4	6	–	–	–	6	–	1	–	5
Juvenile	5	5	10	–	–	–	10	–	1	–	9
Infant	5	3	8	–	–	1	7	–	–	–	8
COLONY 4											
Adult	3	2	1	3	1	4	–	1	4	–	1
Adolescent	2	2	1	2	1	2	1	1	1	1	2
Juvenile	2	0	2	–	–	–	2	–	–	–	2
Infant	1	–	1	–	–	–	1	–	–	–	1

The study site is situated in a miombo woodland forest, an environment ecologically comparable to that of some wild chimpanzee populations (Rawlings et al. 2014). The size of the semi-wild enclosures ranged between 25–77 hectares (Colony 1: 77 ha., total N = 27 members; Colony 2: 65 ha., total N = 57 members; Colony 4: 19 ha., total N = 15 members). Each enclosure contained high trees with grassland underneath, typical for this region. The chimpanzee colonies had similar access to the large numbers of natural objects (sticks, branches, rocks, etc.) and permanent artificial fixtures (water fountains, fences, metal window bars, etc.). Object access extended to non-natural objects that landed accidentally in the enclosures (plastic bottles, drink cans, plastic bags, etc.) and were not necessarily comparable across colonies. In addition to natural barriers such as trees and the distance between the enclosures, artificial fencing is used to separate the colonies. As a result, there was no physical or visual contact between colonies.

Colony compositions were originally organised around the arrival dates of their members rather than their geographical background, which includes a number of different chimpanzee sub-species. Each colony is comprised of a mixture of wild and CWO born chimpanzees. About half of the chimpanzees were wild born who arrived individually or in pairs, of which 24 originated from countries where chimpanzees live naturally. Previous estimates from prior research suggest that in 2011 the subspecies composition depicted 42–65% for *Pan troglodytes schweinfurthii* and 31–42% for *P. t. troglodytes* across the colonies (Rawlings et al. 2014). Importantly, since then and including the period of data collection of this study, there was no addition of new wild chimpanzees to any of the colonies. The rest included individuals who were born in the sanctuary. Due to this mixing of sub-species composition, no apparent sign of phylogenetic differences was evident across the colonies.

Data collection for this study adhered to the criteria of the Ethical Treatment of Nonhuman Primates and Code of Best Practices in Field Primatology outlined by the American Society of Primatologists. Research permits were obtained from the Chimfunshi Research Advisory Board and adhered to the Pan African Sanctuary Alliance research standards. Ethical review was conducted by Animal Welfare and Ethical Review Body of the University of Portsmouth.

### Data collection

An initial period of data collection in August 2012 employed an ad libitum approach (Altmann 1974) where chimpanzees were recorded when they were visible and not sleeping. We recorded individuals across colonies in no particular order or time of the day. This approach incorporated data collection across different social contexts, such as daily feeding, play, grooming, and resting activities. A second period using focal animal sampling method was then conducted between July–September 2013 (Altmann 1974). Here each individual was sampled (randomised and counterbalanced per colony) for 5 min and a record of all individuals within 10 m of the focal was taken. A balance between morning (before 12 p.m.) and afternoon (after 1 p.m.) focal data collection was maintained and subjects were randomly selected each day before data collection. If the focal individual was not visible for more than 5 min, which was possible due the size of the enclosures and the forest density, then the recording focused on the next individual listed for that day. Each day, we attempted to collect two such focal recordings per subject – one in the morning and one in the afternoon. Both collection periods followed the same observational approach. Essentially, the recordings were taken outdoors by an observer standing at ground level within 2–10 m of the chimpanzees. Video data were collected between 7 a.m. and 6 p.m. Across the two data-collection periods a total 1349 video recordings were filmed (ad libitum = Colony 1: 26 h 01 min [156], Colony 2: 16 h 20 min [149], Colony 4: 30 h 31 min [227]; focal = Colony 1: 36 h 24 min [437], Colony 2: 24 h 25 min [293], Colony 4: 7 h 15 min [87], using a PANASONIC HC-V727 full HD and Sony HandyCam DCR-TRV19E cameras.

### Behavioural coding from video

An Object Action occurred when an object was used by a chimpanzee (the subject) during a social interaction with a conspecific. We employed a broad definition in which behaviour could be closely targeted towards an object without physical contact (e.g., peering at close distance), and the use of objects as substrates, including non-resonant substrates where it appeared that they were selected for this action (e.g. stepping on). Two categories of Object Actions were identified: 1) Targeted Object Action: an action in which an object action aimed towards a specific conspecific (e.g., hitting, touching, poking) or made physical contact with them (e.g., throwing at); 2) Nontargeted Object Action: an action in which an object was manipulated or contacted by the subject but not targeting a specific social partner. Since the social factor of the latter may at times be less obvious compared to the Targeted Object Actions, in these events, the social engagement was determined by either the subject and their social partner being in a close social proximity (< 2 m) (e.g., walking or sitting together), showing directed face, and/or by being engaged in an ongoing social interaction (e.g., play chasing). In cases when more than one conspecific was present, the apparent social partner (the recipient) of the interaction was determined by being: in the closest proximity to the subject, and/or face directedness between the two, and/or recent social engagement. We first assessed the orientation of the face (e.g., whether face was directed towards the social partner), followed by the subject’s most recent social interaction (e.g., having played with the social partner), and their subsequent behaviour (e.g., approaching the social partner) to determine the inferred recipient of each object action.

Table 2 lists the 22 types of Object Actions observed in this study, with corresponding definitions and references to previous research.

**Table 2:** Definitions of targeted and nontargeted object actions observed in the chimpanzees of the current study and an overview of where comparable object actions were previously reported as part of the great ape gestural communication repertoire

Object actions	Definition	
Targeted object actions		
Hitting	Bringing an object into a short hard contact with the recipient	^a,b,d^
Hitting threat	Performing a hit move towards recipient but without coming into contact with their body	^a,c^
Poking	Jabbing another’s body with an object	^a^
Pulling	Pulling the object from another	^a,b,c^
Showing	Presenting an object so the other one could see it	^a,c^
Spitting water	Spitting water towards another	^a^
Throwing at	Throwing an object towards recipient or another direction	^a,b,c,d^
Throwing threat	Performing a throw move towards another but without releasing the object	^a,d^
Touching	Bringing an object in a gentle contact with the body of another	^a^
Nontargeted object actions		
Carrying/holding	Picking up and carrying/holding an object	^a,b^
Peering	Closely examining an object with eyes	^a^
Manufacturing	Reshaping an object e.g. peeling off the layers, breaking, or bending	^a,b^
Moving	Displacing an object from one place to another	^a,b,c,d^
Object in mouth	Placing an object in the mouth without chewing/swallowing it	^a,b^
Self-grooming	Using an object to groom/scratch oneself	^a^
Shaking	Repeatedly moving an object back and forth	^a,b,c,d^
Slapping	Slapping an object with the palm of its hands or the feet	^a,b,d^
Splashing water	Moving the hand through a water surface	^a,b,c,d^
Stepping	Stepping on the object with its feet	^b,d^
Tapping	Using the fingers to come into a short contact with an object	^a,b,d^
Throwing around	Throwing an object into the air (not at the recipient)	^a,b,c,d^
Waving	Waving an object above the head or around its body	^a,d^

^a^*Chimpanzees*: Fröhlich et al. (2017); Hobaiter and Byrne (2011b); Koops et al. (2015); Liebal et al. (2004a); Nishida et al. (1999); Pollick and de Waal (2007);

Roberts et al. (2019); Roberts et al. (2014); Schneider et al. (2012); Silk et al. (2013); Tomasello et al. (1994)

^b^*Bonobos*: Graham et al. (2017); Graham et al. (2018); Koops et al. (2015); Nishida et al. (1999); Pika et al. (2005); Pollick and de Waal (2007); Schneider et al.(2012)

^c^*Orangutans*: Liebal et al. (2006); Schneider et al. (2012)

^d^*Gorillas*: Genty et al. (2009); Pika et al. (2003); Schneider et al. (2012)

For every Object Action, the following was coded:

1. Behavioural contexts of the subject. The behavioural context referred to the situational circumstances during which the onset of the object action was observed. Seven mutually exclusive categories were used: social play, solitary play, allogrooming, autogrooming, feeding, resting, and agnostic and (see Table 3 for definitions) (Chotard et al. 2023; Liebal et al. 2006; Pika et al. 2005). In cases where the context of the object action changed mid event (e.g., prolonged touching), we coded the initial context during which the object action was instigated.
2. Object type, material, and category. Three categories of object material rigidity were defined based on the object’s response to force applied by the contact of the chimpanzee’s hand (seeFeix et al. 2014; Rocha et al. 2006). These categories related to the object’s stiffness, bendability, and malleability. The three categories included: (a) soft e.g. a grass straw (b) medium e.g. a bendy stick, and (c) hard e.g. a rock. A further distinction was made between natural (14 items e.g. stick, branch, stone) and artificial (9 items e.g. drink can, plastic bag, fabric strip) objects (see Table 4 for definitions).
3. Face directedness. Face directedness referred to the orientation of the social partners’ and subjects’ faces two seconds before each Object Action (as in: Taylor et al. (2022) and was coded as a categorical variable (face directed vs face nondirected). Face directed was coded when the subject’s face was oriented towards the conspecific and judged as oriented to be within < 45◦ of a direct line to the centre of the social partner’s face, and the social partner was not facing away from the subject. This also included mutual face directedness. Face nondirected was coded when the subject’s face was oriented towards the conspecific and judged as oriented to be > 45◦ of a direct line to the centre of the social partner’s face or when the social partner was facing fully away from the subject.
4. Recipient’s behaviour change. The alteration in the recipient’s behaviour was coded as a categorical variable (change vs no change) see: Liebal et al. (2006); Pika et al. (2005). We measured changes in the recipients’: (a) face directedness towards the subject as indicated by the head movement (yes vs no), (b) movement with respect to the subject’s location (approach, leave, no change), and change in behavioural context (yes vs no) 2 s before and 2 s after each Object Action.

**Table 3:** Definitions of behavioural contexts and their corresponding valence

Context type and valence	Definition	Occurrence of actions [% (frequency)]	
		Targeted	Nontargeted
Positive valence			
Social play	Playful activity and/or interactions between conspecifics	23.93 (75)	18.04 (94)
Solitary play	Playful activity by oneself	26.79 (67)	43.95 (229)
Allogrooming	Social grooming, conspecifics look through others’ hair	1.43 (4)	1.54 (8)
Neutral valence			
Autogrooming	Solitary self-grooming	0.71 (2)	0.77 (4)
Feeding	Extractive foraging and eating	20.36 (57)	7.10 (37)
Resting	Sitting or lying down	20.00 (56)	26.49 (138)
Negative valence			
Agnostic	Threatening/dominating displays e.g. charging, chasing, physical violence	6.79 (19)	2.11 (11)

**Table 4:** Summary of object types used by chimpanzees in targeted and nontargeted object actions, their rigidity, description, and occurrence

Object rigidity and type	Description	Number of occurrences
		Targeted | Nontargeted
Soft		
Food	Any plant part that is consumed as food by chimpanzees, including mostly fleshy fruits and vegetables**	76 | 33
Grass straw	Dry stems of plants e.g. grass	11 | 38
Plastic bag*	Thin, lightweight plastic sack	14 | 16
Water	Drinking water from a fountain or pond	7 | 11
Pebble-like	Pebble-sized natural object found on the ground	5 | 11
Tissue paper*	Thin, lightweight sheet of tissue paper	5 | 7
Hay	Dry grass and plants, attached/unattached to the ground	2 | 6
Leaf	Plant leaf unattached to a tree/plant	0 | 4
Plant	Outdoor plant attached/unattached to the ground e.g. shrub	0 | 2
Rope*	Set of fibre strands twisted together into a long cord shape	0 | 1
Medium		
Stick	Stick attached/unattached to a tree (chimp-finger thick/thicker)	15 | 66
Plastic bottle*	Empty slender plastic container used to store liquids	33 | 43
Tree bark	Outer layer of tree trunk and branches (chimp arm-sized/lager)	25 | 40
Fruit peel	Empty fruit shells of *Strychnos spinosa*	18 | 28
Plastic tube*	Long plastic cord with hollow inside	5 | 10
Dead Animal	Small animal carcass	3 | 6
Hard		
Tree branch	Branch attached/unattached to a tree (chimp-arm length/longer)	41 | 103
Cement block*	Concrete block similar in size to a construction brick	13 | 79
Drink can*	Empty aluminium beverage can	4 | 6
Rock	Hard, solid rock (chimp fist/bigger)	1 | 5
Stone	Hard, solid stone (smaller than chimp fist)	2 | 1
Fountain*	Rectangular cement water column with a protruding spout	0 | 2
Ground	Ground/floor element of the enclosure	0 | 2
Metal bars*	Metal bars attached to windows of the enclosure buildings	0 | 1

^*^Artificial, manufactured objects

^**^The events in which food was used were limited to soft and malleable fruits and vegetables

To address pseudoreplication (Hurlbert 1984), if an object action was repeated within 3 s of the previous one, it was considered to be part of the same event series. If, however, the object action incorporated a different object, was targeted at a different recipient, or followed after 3 s of the previous object signal, it was coded as a separate event. For example, if a subject touched the same recipient with the same stick twice within 3 s it was counted as a single event series of Object Actions. In total, 895 series (364 targeted, 531 nontargeted) were coded.

Video recordings were analysed by one main coder (VG) who was naïve about the hypotheses of this study at the time of the coding using InterAct Mangold Lab Suite Version 2015 (Program Version 15.0.0.0—Arnstorf, Germany; 25 f.p.s.). Inter-coder reliability was tested on 15% of object actions by a second coder using Cohen’s Kappa (Cohen 1960). High levels of agreement (Fleiss et al. 2003) were found for the types of Object Actions (κ = 0.91), behavioural contexts (κ = 0.89), and face directedness (κ = 0.83).

### Statistical analyses

We first addressed the use of objects by describing the total number of Object Actions produced by all 74 subjects during social interactions, and in which behavioural contexts these occurred. To assess the effect of different features of object actions as well as subjects’ characteristics on social interactions, we used two generalized linear mixed models (GLMM) with a binomial error structure. In the first two models the dependent variable was the occurrence of targeted object actions. Model 1 estimated the effect of colony membership and an interaction effect [face directedness* action modality] on whether the subject produced a targeted object action. Model 2 estimated the effect of colony membership and an interaction effect [face directedness*object rigidity] on whether the subject produced a targeted object action. Including an interaction effect into the model helped to examine how the effect of directedness on the production of targeted object actions may differ depending on sensory modality of the action and the object rigidity. Initially, subjects’ age group was included as a predictor. However, when age group was included, both models were unable to converge. It is likely that the models were too complex relative to the available data to estimate the model parameters with sufficient accuracy. Since, the aim of the analyses was to examine the effect of specific interactions we decided to remove the age group predictor from the final models. The third and final model (Model 3) estimated the effect of four predictors, namely the object action type, face directedness, modality of the action, and subject’s age group on whether a social partner changed their behaviour following an object action. Since we had multiple data points for each subject with different number of observations, the subject ID was used as a random factor to account for the variance between the chimpanzees. All models successfully converged and were compared using the likelihood ratio test. All models were evaluated and met the assumptions of normality of residuals, homogeneity of variances, and absence of multicollinearity. Wald Chi- Square with Confidence Intervals (CI) of 95% were used to assess each parameter in the final models. Marginal R^2^ values were calculated for both models (Nakagawa and Schielzeth 2013). No cases of missing data were reported. The analysis was carried out using RStudio v2023.03.0 + 386 (RStudio, Boston, MA, USA) using the ‘(g)lmer’ function (‘lme4’ and ‘optimix’ packages).

## Results

Twenty-two distinct Object Action types were observed in the study (see Table 2). These included 10 Targeted Object Actions (280 occurrences) and 13 Nontargeted Object Actions (521 occurrences).

### Flexibility to behavioural context

Chimpanzees employed Object Actions in all observed behavioural contexts. All Object Action types observed in the study occurred in more than one context. Table 5 shows the different behavioural contexts in which each Object Action occurred, where both Targeted and Nontargeted Object Actions were found in up to 6 behavioural contexts (range 2–6, mean = 4.26, SD = 1.25). Two Targeted Object Action types: showing (n = 51 occurrences) and touching (n = 12); and two Nontargeted Object Action types: holding/carrying (n = 132), and object in mouth (n = 77), were observed across all 6 contexts.

**Table 5:** The total frequency of each object action type and behavioural contexts in which they occurred. Altogether 22 types of object actions were identified based on 801 occurrences

Object actions	Sum of contexts (Frequency of actions*)*	Types of contexts (Frequency of actions for each context category)
Targeted object actions		
Showing	6 (51)	Solitary play (15), social play (14), resting (15), feeding (4), allogrooming (2), autogrooming (1)
Touching	6 (12)	Solitary play (2), social play (5), resting (2), feeding (1), allogrooming (1), autogrooming (1)
Pulling	5 (102)	Solitary play (8), social play (23), resting (29), feeding (41), agnostic (1)
Hitting	5 (41)	Solitary play (17), social play (10), resting (6), feeding (1), agnostic (7)
Throwing at	5 (37)	Solitary play (16), social play (9), resting (3), feeding (2), agnostic (7)
Hitting threat	5 (13)	Solitary play (7), social play (2), resting (1), feeding (2), agnostic (1)
Poking	3 (11)	Solitary play (7), social play (3), allogrooming (1)
Spitting water	2 (7)	Social play (1), feeding (6)
Throwing threat	2 (6)	Solitary play (3), agnostic (3)
Showing	6 (51)	Solitary play (15), social play (14), resting (15), feeding (4), allogrooming (2), autogrooming (1)
Nontargeted object actions		
Holding/carrying	6 (132)	Solitary play (45), social play (22), resting (50), feeding (7), agnostic (5), allogrooming (3)
Object in mouth	6 (77)	Solitary play (36), social play (19), resting (13), feeding (7), agnostic (1), allogrooming (1)
Moving	5 (62)	Solitary play (33), social play (3), resting (15), feeding (9), allogrooming (2)
Throwing around	5 (16)	Solitary play (8), social play (5), resting (1), feeding (1), agnostic (2)
Waving	5 (121)	Solitary play (65), social play (33), resting (18), feeding (4), agnostic (1)
Peering	4 (41)	Solitary play (11), resting (21), feeding (8), autogrooming (1)
Reshaping	4 (14)	Solitary play (1), social play (3), resting (9), autogrooming (1)
Self-grooming	4 (5)	Social play (1), resting (1), allogrooming (1), autogrooming (2)
Shaking	4 (8)	Solitary play (4), social play (2), resting (1), feeding (1)
Stepping	4 (15)	Solitary play (10), social play (3), agnostic (1), allogrooming (1)
Slapping	3 (4)	Solitary play (1), resting (2), agnostic (1)
Splashing	3 (20)	Solitary play (12), social play (2), resting (6)
Tapping	3 (5)	Solitary play (3), social play (1), resting (1)

Most Targeted Object Actions were observed during social play (n =75 occurrences), solitary play (n = 67), and feeding (n = 57), whereas most Nontargeted Object Actions were reported during solitary play (n = 229), resting (n = 138) and social play (n = 94) (see Table 3). The analysis revealed a positive correlation between the number of Object Actions produced by the subjects and the number of contexts in which they were observed (two-tailed Pearson's Product–Moment, N = 75, r(22) = 0.43, p < 0.001, 95% CI [0.26, 0.62]).

### Flexibility in targeted object actions

A full model (Model 1) that predicted the occurrence of targeted Object Actions (AIC: 829.59, BIC: 871.76) fit the data significantly better than the null model (AIC: 1004.48, BIC: 1013.85; χ^2^(10) = 188.89, p < 0.001). The full model was also significantly different to a reduced model without the interaction term (AIC: 847.80, BIC: 880.60) and indicated a better fit (χ^2^(2) = 22.21, p < 0.001). The proportion of variance attributed to the fixed effects in Model 1 was R2 = 0.41. The variance inflation factor (VIF) was used to estimate collinearity between the fixed factors. As a general rule the VIF should not exceed 10 (Robinson and Schumacker 2009; Vittinghoff 2005), which was the case in the full model which indicated a low correlation (VIF: 1.02 – 4.68). Overdispersion measures of the model showed no significant violations (χ2 = 663.76, p = 0.998). The relationship between the fitted values from the model and the simulated residuals suggested that the full model adequately captured the variation in the data. Comparing the distribution of the simulated residuals to a normal distribution showed no violations of the simulated residuals. The variance estimates of random effects was 0.75 (SD = 0.87).

**Fig. 1:**
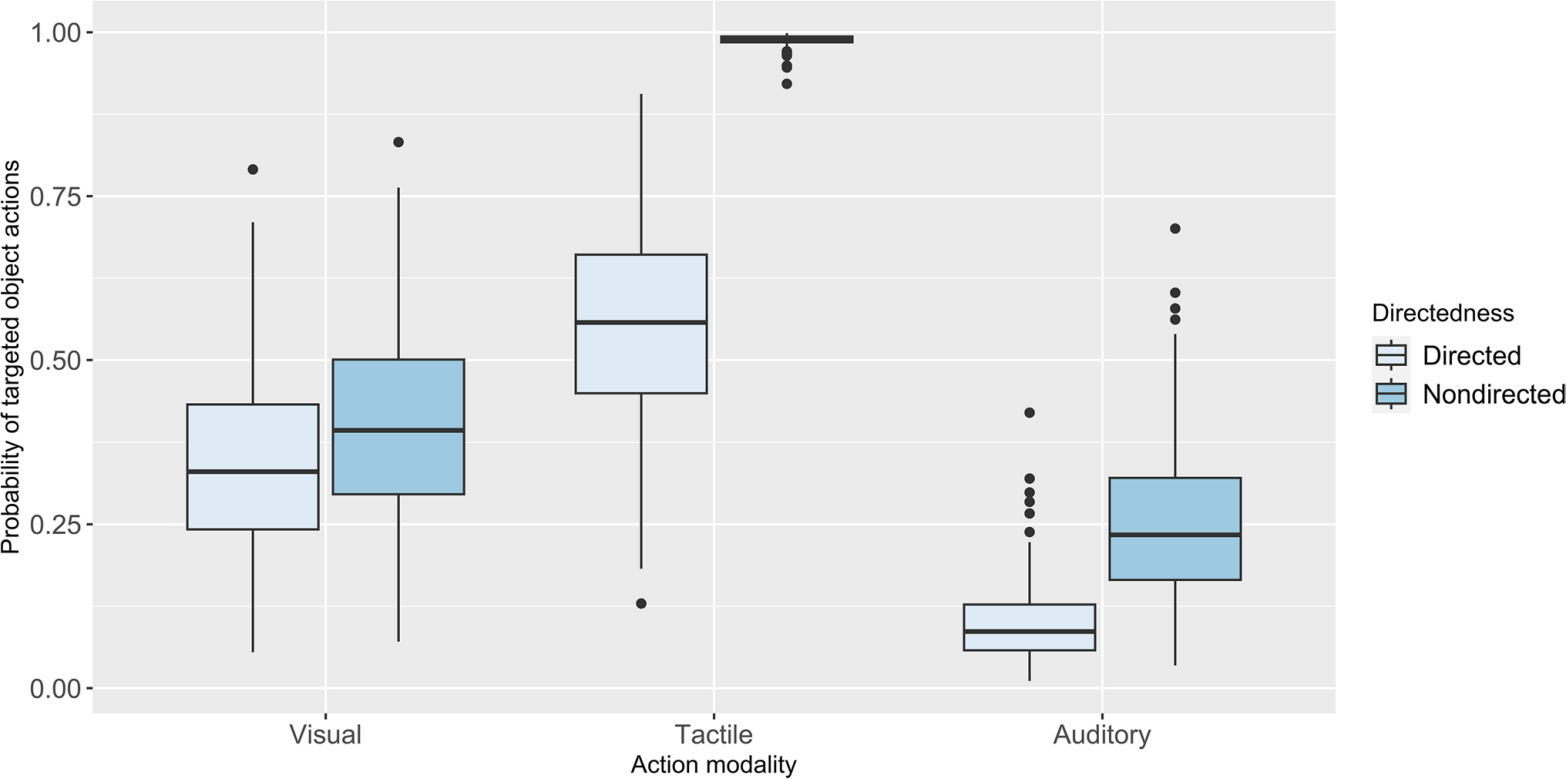
Main effects of interaction using face directedness* action modality as predictors of targeted Object Actions. The light blue boxes represent face directed and dark blue boxes face nondirected. The thick horizontal lines depict medians, and the thin lines maximum and minimum range values. The upper and lower quartiles are indicated by the box lengths. The vertical lines indicate 95% confidence intervals

In the full model, several significant effects related were found with targeted Object Actions significantly more likely to occur during tactile (estimate ± se = 2.59 ± 0.60, p < 0.001) and visual (estimate ± se = 1.65 ± 0.33, p < 0.001) object actions, and when chimpanzees showed nondirected face (estimate ± se = 1.17 ± 0.36, p = 0.001). However, there was also a significant interaction between face directedness and modality of the action. Targeted Object Actions were significantly more likely to occur during tactile object actions when not facing (estimate ± se = 3.20 ± 1.22, p = 0.009), but less likely to occur during visual object actions when not facing (estimate ± se = -0.90 ± 0.42, p = 0.034) (see Fig. 1).The analysis showed a significant main effect associated with the subject’s colony membership. Specifically, it was found that targeted Object Actions were more likely to occur among the members of Colony 2 (estimate ± se = 0.84 ± 0.40, p = 0.035). All model values for the full Model 1 are shown in Table 6.

**Table 6:** Features of, colony membership and face directedness*action modality as predictors of targeted object actions (n = 75)

Predictors	*B* (SE)	95% Wald confidence interval for (*B)*		Wald chi-square (*df*)	*z value*	*p* value
		Lower	Upper			
(Intercept)	−2.81 (0.45)	−3.70	−1.92	0.00	−6.19	0.000
Colony: one	0.41 (0.43)	−0.43	1.24	0.34	0.95	0.340
Colony: two	0.84 (0.4)	0.06	1.62	0.04	2.11	0.035
Face: nondirected	1.17 (0.36)	0.47	1.87	0.00	3.28	0.001
Modality: tactile	2.59 (0.6)	1.41	3.77	0.00	4.30	0.000
Modality: visual	1.65 (0.33)	1.00	2.30	0.00	5.00	0.000
Nondirected*Tactile	3.2 (1.22)	0.81	5.58	0.01	2.63	0.009
Nondirected *Visual	−0.9 (0.42)	−1.73	−0.07	0.03	−2.12	0.034

A full model (Model 2) that predicted the occurrence of targeted Object Actions (AIC: 922.18, BIC: 965.06) fit the data significantly better than the null model (AIC: 1004.48, BIC: 1013.85; χ^2^(7) = 107.3, p < 0.001). This full model was also significantly different to a reduced model without the interaction term (AIC: 966.48, BIC: 999.28) and indicated a better fit (χ^2^(2) = 47.60, p < 0.001). The full model was therefore selected. The proportion of variance attributed to the fixed effects in Model 1 was R2 = 0.17. The variance inflation factor (VIF) indicated a low correlation for fixed factors correlation (VIF: 1.03–4.94) and high for the interaction term (VIF = 12.05). To investigate this further, we computed a correlation matrix to assess the pairwise correlations among the predictor variables and found no notable violations in the colleniality between the variables. Overdispersion measures of the model showed no significant violations (χ2 = 755.50, p = 0.820). The relationship between the fitted values from the model and the simulated residuals suggested that the full model adequately captured the variation in the data. Comparing the distribution of the simulated residuals to a normal distribution showed no violations of the simulated residuals. The variance estimates of random effects was 0.52 (SD = 0.72).

**Fig. 2:**
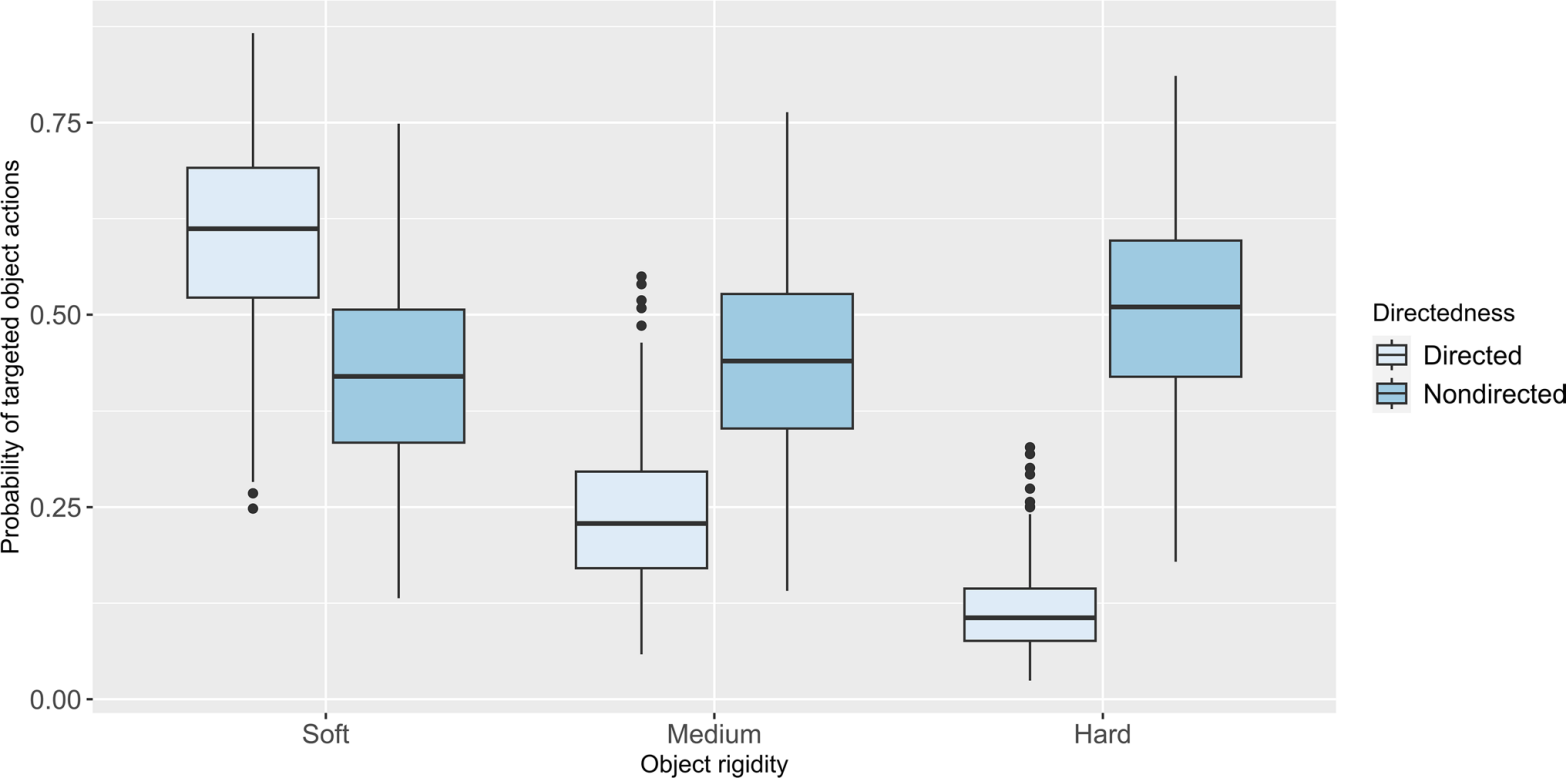
Main effects of interaction using face directedness* object rigidity as predictors of targeted object actions. The light blue boxes represent face directed and dark blue boxes face nondirected. The thick horizontal lines depict medians, and the thin lines maximum and minimum range values. The upper and lower quartiles are indicated by the box lengths. The vertical lines indicate 95% confidence

In the full model, we found a significant effect of face directedness with targeted Object Actions significantly more likely to occur during when chimpanzees showed face nondirected (estimate ± se = 2.17 ± 0.35, p < 0.001) and when using objects of soft (estimate ± se = 2.59 ± 0.34, p < 0.001) and medium (estimate ± se = 0.92 ± 0.33, p = 0.005) rigidity. There was also a significant interaction between face directedness and rigidity of the object. Targeted Object Actions involving soft (estimate ± se = -2.95 ± 0.45, p < 0.001), or medium (estimate ± se = -1.20 ± 0.45, p = 0.007) were less likely to occur during nondirected face (see Fig. 2). All model values for the full Model 2 are shown in Table 7.

**Table 7:** Features of colony membership and face directedness*object rigidity as predictors of targeted object actions (n = 75)

Predictors	*B* (SE)	95% Wald confidence interval for (*B)*		Wald chi-square (*df*)	*z value*	*p* value
		Lower	Upper			
(Intercept)	−2.54 (0.39)	−3.3	−1.78	43.30	−6.58	0.000
Colony: one	0.53 (0.38)	−0.21	1.26	1.96	1.40	0.162
Colony: two	0.65 (0.35)	−0.04	1.34	3.43	1.85	0.064
Face: nondirected	2.17 (0.35)	1.48	2.86	38.28	6.19	0.000
Rigidity: soft	2.59 (0.34)	1.93	3.25	58.76	7.67	0.000
Rigidity: medium	0.92 (0.33)	0.28	1.56	7.90	2.81	0.005
Nondirected*Soft	−2.95 (0.45)	−3.84	−2.06	42.44	−6.52	0.000
Nondirected *Medium	−1.2 (0.45)	−2.08	−0.32	7.15	−2.67	0.007

### Flexibility to recipient’s behaviour

A full model in which the change in the recipient’s behaviour was predicted by Object Action type, face directedness, modality of the action, and subject’s age group AIC: 995.45, BIC: 1040.60) was significantly different from a null model (AIC: 1055.09, BIC: 1064.46; χ^2^(7) = 70.64, p < 0.001). The proportion of variance attributed to fixed effects was R2 = 0.14. The variance inflation factor (VIF) showed a low correlation (VIF: 1.08 – 1.23). Overdispersion measures of the model showed no significant violations (χ^2^ = 749.07, p = 0.860). The relationship between the fitted values from the model and the simulated residuals showed that the model adequately captured the variation in the data. Comparing the distribution of the simulated residuals to a normal distribution showed no violations of the simulated residuals. The variance estimates of random effects was 0.14 (SD = 0.37).

In the full model, a significant main effect of Object Action was found with changes in the recipients’ behaviour significantly more likely to occur following targeted Object Actions compared to non-targeted Object Actions (estimate ± se = 0.75 ± 0.20, p < 0.001). Changes in the recipient’s behaviour were also more likely to follow Object Actions that were produced when chimpanzees were not face directed (estimate ± se = 0.77 ± 0.17, p < 0.001). There was also a significant main of the age group where changes in the recipient’s behaviour were less likely to occur following Object Actions that were produced by infant chimpanzees (estimate ± se = -0.98 ± 0.34, p = 0.003). In the full model, there was no significant main effect of modality. All model values for the full Model 3 are shown in Table 8. For the observed types of recipients' behavioural changes for each type of Object Action, please see Table 9.

**Table 8:** Features of object action type, face directedness, action modality, and subjects’ age group as predictors of changes in the recipients’ behaviour (n = 75)

Predictors	*B* (SE)	95% Wald confidence interval for (*B)*		Wald chi-square (*df*)	*z value*	*p* value
		Lower	Upper			
(Intercept)	0.42 (0.30)	−0.16	1.00	2.05 (1)	1.43	0.152
Action type: targeted	0.75 (0.20)	0.37	1.13	14.72 (1)	3.84	0.000
Face: nondirected	0.77 (0.17)	0.43	1.11	19.85 (1)	4.46	0.000
Modality: tactile	0.54 (0.35)	−0.15	1.23	2.38 (1)	1.54	0.123
Modality: visual	0.03 (0.17)	−0.31	0.37	0.02 (1)	0.15	0.888
Age group: infant	−0.98 (0.34)	−1.64	–0.32	8.45 (1)	–2.91	0.004
Age group: juvenile	−0.41 (0.31)	−1.02	0.20	1.72 (1)	−1.31	0.190
Age group: adult	−0.34 (0.31)	−0.95	0.27	1.18 (1)	−1.09	0.277

**Table 9:** The total frequency of each object action within each category, number of subjects and percentage distribution with which each object action evoked a change in recipients’ behaviour

Object action	Change in behaviour (%)		Sum of change types (Frequency of actions*)*	Types of changes (Frequency of actions for each category)	
	Yes	No			
Targeted object actions					
Pulling	66.67	33.33	7 (68)	Facing (29), movement (20), context (2), facing & movement (8), facing & context (2), movement & context (5), all (2)	
Showing	54.90	45.10	7 (28)	Facing (6), movement (11), context (1), facing & movement (5), facing & context (1), movement & context (2), all (2)	
Hitting	92.68	7.32	6 (38)	Facing (21), movement (4), facing & movement (8), facing & context (2), movement & context (1), all (2)	
Throwing at	97.30	2.70	5 (36)	Facing (20), movement (2), facing & movement (7), facing & context (5), movement & context (2)	
Hitting threat	100.00	0.00	5 (13)	Facing (6), movement (4), facing & movement (1), movement & context (1), all (1)	
Touching	75.00	25.00	4 (9)	Facing (6), context (1), facing & movement (1), facing & context (1)	
Throwing threat	83.33	16.67	4 (5)	Facing (2), movement (1), facing & movement (1), all (1)	
Spitting water	100.00	0.00	3 (7)	Facing & movement (3), movement & context (3), all (1)	
Poking	100.00	0.00	2 (11)	Facing (10), all (1)	
Nontargeted object actions					
Holding/carrying	56.82	43.18	7 (75)	Facing (21), movement (25), context (6), facing & movement (6), facing & context (7), movement & context (9), all (1)	
Waving	46.28	53.72	7 (56)	Facing (11), movement (22), context (5), facing & movement (7), facing & context (2), movement & context (8), all (1)	
Object in mouth	54.55	45.45	6 (42)	Facing (10), movement (18), context (6), facing & movement (2), facing & context (3), movement & context (3)	
Moving	50.00	50.00	6 (31)	Facing (11), movement (8), context (3), facing & movement (2), facing & context (1), movement & context (6)	
Throwing around	88.24	11.76	6 (15)	Facing (9), context (2), facing & movement (1), facing & context (1), movement & context (1), all (1)	
Peering	56.10	43.90	5 (23)	Facing (2), movement (14), context (1), facing & movement (5), all (1)	
Splashing	95.00	5.00	5 (19)	Facing (12), movement (4), facing & movement (1), facing & context (1), movement & context (1)	
Stepping	26.67	73.33	4 (4)	Facing (1), context (1), movement & context (1), all (1)	
Shaking	87.50	12.50	4 (7)	Facing (2), movement (1), context (2), facing & movement (2)	
Reshaping	57.14	42.86	3 (8)	Facing (5), movement (2), movement & context (1)	
Tapping	60.00	40.00	3 (3)	Facing (1), movement (1), facing & movement (1)	
Slapping	50.00	50.00	2 (2)	Movement (1), context (1)	
Self-grooming	40.00	60.00	1 (2)	Facing (2)	

## Discussion

This study examined object actions (i.e., the use of an object during a social interaction) shown by semi-wild chimpanzees during social interactions. Altogether twenty-two types of object actions were found and all types occurred in more than one behavioural context, suggesting flexibility in the use of object actions across contexts. Of the 22 types of object actions, 10 were considered to target a specific recipient. These targeted actions evoked substantially greater response rates, were adjusted to the attentional state of the recipient, and showed apparent colony differences. This pattern of results highlights the interactive features of targeted object actions and their use in nonvocal communication. Chimpanzees incorporate a wide range of object actions in their communication, these show high flexibility and show the potential to alter the behaviour of the recipient.

Given the evidence that chimpanzee gestures are used in a flexible manner by occurring, for instance, across behavioural contexts (Call and Tomasello 2007; Hobaiter and Byrne 2011a), we proposed that object actions would show similar patterns of contextual flexibility. Indeed, supporting this hypothesis all object actions were observed in at least two behavioural contexts, where actions such as showing and touching were found across all seven contexts. These findings suggest that specific types of object interactions are not bound to circumstances imposed by the behavioural context and that the chimpanzees of this study showed the ability to flexibly incorporate communicative actions that involve objects across various social interactions. The indication that chimpanzees have the capacity to use communicative object actions flexibly across different situational contexts expands our current understanding, which has been largely limited to gesture research. We suggest that the cognitive abilities currently linked to the flexible use of gestures, seem to extend to the broad use of communicative interactions with objects. We found a sampling effort effect that suggested the observed flexibility of object actions was limited by the number of observations, rather than chimpanzees' use of them, suggesting further observations would reveal greater flexibility. Extending the observation that chimpanzees use gestures flexibly (Call and Tomasello 2007; Hobaiter and Byrne 2011a; Tomasello et al. 1994), our findings suggest object actions possess similar characteristics. We argue that the ability of chimpanzees to produce distinctive object actions independently of behavioural context extends the versatility present in ape communication. This flexibility suggests that object signals, similarly to gestures, may have contributed to establishing the groundwork required for the emergence of flexible characteristics in human language.

While selection of object signals can vary within a context our findings also point to the effect of face directedness on the occurrence of targeted object actions. More specifically, silent visual object signals appear to be produced less often during nondirected facing, compared to tactile object actions, which occurred more often when nonface directed (Canteloup et al. 2015; Genty et al. 2009; Liebal et al. 2006; Tomasello et al. 1994). Inclusion of physical objects in communicative interactions can introduce an audible component that, in some cases, may replace the use of vocal signals to obtain the recipient’s attention or transmit information to non-visually attentive recipients. However, the form of such signals is dependent on the acoustic properties of the object type, whilst vocalisations may offer greater independence from the immediate environment. Nevertheless, the use of silent visual actions when the recipient is visually attentive not only indicates sensitivity to the attentional states of others, but perhaps also provides the option to communicate with specific audiences and not others (Hare et al. 2001; Leavens et al. 2004b; Schel et al. 2013). For example, silent object showing allows the signaller to display the object to a particular individual without bringing it to the attention of others (Tanner and Byrne 2010). Doing so could be beneficial when building relationships with specific group members by, for instance, sharing resources discreetly or initiating play with preferred partners (Davila-Ross and Dezecache 2021). The ability to adjust communicative object use to the attentional states of others adds a novel dimension to the multi-modal nature of ape communication, where object actions may replace or be used alongside with other signals (Oller and Griebel 2008). Such sensory manipulation was, notably, not supported by more recent studies (Botting and Bastian 2019; Poss et al. 2006; Tempelmann et al. 2011). However, factors such as the presence of objects, naturalistic setting, and the ecological approach used in the current study may have contributed to the differences in our findings.

Sensitivity to the attentional states of others was also observed in the choice of objects. Chimpanzees used objects of soft and medium rigidity less frequently, compared to hard objects, during interactions in which they were not face-to-face with their recipient. This selectivity demonstrates that chimpanzees may select the specific physical properties of objects not only in strictly practical applications, such as tool use (Sanz et al. 2004), but also in their social and communicative behaviours. A recent study on wild chimpanzee buttress drumming suggested that chimpanzees select particular tree buttresses on the basis of their resonant acoustic properties (Fitzgerald et al. 2022). It indicates that chimpanzees may be sensitive to objects’ distinctive acoustic and tactile properties, and the way in which they may impact their use in communication. Further research is, however, needed to examine whether chimpanzees use specific techniques of object interaction to first establish visual contact with the recipient–i.e. as ‘visual attention getters’. There is some evidence for their use in wider behaviour to adjust attention in gestural communication (Liebal et al. 2004a) but this concept is yet to be examined in relation to object based actions.

In addition, cross-colony comparisons were carried out. The results showed that the occurrence targeted object action was higher in Colony 2. However, these findings should be interpreted with caution as a significant effect was also found the sensory modality of the object actions. This effect could be explained by some targeted object actions such as touching, hitting, and poking being exclusively tactile, which was not the case for any of the nontargeted object actions. Nevertheless, since self-grooming and spitting were limited to specific colonies, and knowing that some gestures may be socially learned (Pika et al. 2003; Badihi et al. 2023), our findings suggest that there may be promise in further investigation of colony differences in specific object action types. When doing so, consideration should be extended to the fact that differences in group dynamics and social density may lead to unequal opportunities for socialisation across colonies (Cronin et al. 2014; Rawlings et al. 2023). For example, in this study, Colony 2 had a larger number of members as compared to Colony 1.

In summary, this study provides evidence that chimpanzees not only use objects in their broader social interactions but also use them in targeted ways to communicate with conspecifics. The use of object actions in communication appears to follow a similar pattern to that reported for gestural communication, with chimpanzees showing sensitivity to the attentional states of others, as well as showing potential colony differences. While descriptions of object-based gestures were previously reported in research, the present study provides the first direct exploration of communicative object-based signals expanding our understanding of nonvocal communication beyond traditional gestures and suggesting that the extent of object-based signals may be more prevalent that initially suspected. We argue that by expanding the use of objects outside of practical settings, such as resource extraction, social object actions may have supported to the development of manual praxis, similarly to tool use (Arbib 2011; Ruck 2014), providing a scaffold for gestural communication. The sensitivity to the recipient’s visual attention suggests that a certain degree of order and planning may already be involved in object actions, representing a potential first step in the evolution of apes’ rich system of manual gestural communication.

## Data Availability

Data are available from the corresponding author on reasonable request.
